# Supplementary material for: Long-term follow-up of applying autologous bone grafts for reconstructing tympanomastoid defects in functional cholesteatoma surgery
Source: PeerJ. 2021 Nov 23;9:e12522. doi: 10.7717/peerj.12522 (PMC8621709; doi:10.7717/peerj.12522)
Supplement: Supplemental Information 2 [file peerj-09-12522-s002.docx]

| **Code** | **Factor** | **Convert** |
| --- | --- | --- |
| age | age |  |
| age_ | age distribution | 0=<=45 years  1= 45 years |
| sex | gender | Female=0, Male=1 |
| ear | OP ear | 0=right 1=left |
| preOP | Previous ear surgery | 0=no, 1=yes |
| HTN | HTN | 0=no, 1=yes |
| DM | DM | 0=no, 1=yes |
| AR | Allergic rhintis | 0=no, 1=yes |
| SMO | Smoking | 0=no, 1=yes |
|  |  |  |
| PreAC_0.5K | Pre OP 500 Hz in AC |  |
| PreAC_1K | Pre OP 1000 Hz in AC |  |
| PreAC_2K | Pre OP 2000 Hz in AC |  |
| PreAC_4K | Pre OP 4000 Hz in AC |  |
| Pre_PTA | Average of pre AC, 0.5k-4k Hz |  |
| Pre_PTA_ | PTA distribution | 0=≤40 dB  1=>40 dB |
| PreBC_0.5K | Pre OP 500 Hz in BC |  |
| PreBC_1K | Pre OP 1000 Hz in BC |  |
| PreBC_2K | Pre OP 2000 Hz in BC |  |
| PreBC_4K | Pre OP 4000 Hz in BC |  |
| PreABG_0.5K | Pre-OP ABG at 0.5K |  |
| PreABG_1K | Pre-OP ABG at 1K |  |
| PreABG_2K | Pre-OP ABG at 2K |  |
| PreABG_4K | Pre-OP ABG at 4K |  |
| Pre_ABG | Average of pre ABG, 0.5k-4k Hz |  |
| PostAC_0.5K | post OP 500 Hz in AC of latest records |  |
| PostAC_1K | post OP 1000 Hz in AC of latest records |  |
| PostAC_2K | post OP 2000 Hz in AC of latest records |  |
| PostAC_4K | post OP 4000 Hz in AC of latest records |  |
| Post_PTA | Average of post AC, 0.5k-4k Hz of latest records |  |
| PostBC_0.5K | post OP 500 Hz in BC of latest records |  |
| PostBC_1K | Post OP 1000 Hz in BC of latest records |  |
| PostBC_2K | Post OP 2000 Hz in BC of latest records |  |
| PostBC_4K | Post OP 4000 Hz in BC of latest records |  |
| PostABG_0.5K | Post-OP ABG at 0.5K of latest records |  |
| PostABG_1K | Post-OP ABG at 1K of latest records |  |
| PostABG_2K | Post-OP ABG at 2K of latest records |  |
| PostABG_4KS | Post-OP ABG at 4K of latest records |  |
| Post_ABG | Average of post ABG, 0.5k-4k Hz of latest records |  |
| PTA_imp | prePTA - postPTA |  |
| ABG_imp | preABG - postABG |  |
| success | (1) no recurrent or residual cholesteatoma during the follow-up period and (2) gain of at least 15 dB in the PTA when compared with preoperative data or conservation of the hearing value in patients with normal preoperative hearing (PTA ≤ 25 dB) | 0=no success  1=success |
| Site and stage | site(and extension of cholsteatoma)  stage I: one site  stage II: more than two sites  stage III: a with extracranial complications and/or intratemporal pathologic conditions［Facial palsy, , labyrinthine fistula, canal wall destruction, adhesive otitis, , petrous bone or skull base destruction, neck abscess ]  stage IV: with intracranial complications[Purulent meningitis, epidural abscess, subdural abscess, brain abscess, sinus thrombosis] | site:  1=attic only (pars flaccida) 2=mesotympanum only (pars tensa)  3=attic with extension to antrum/mastoid  4=mesotympanum with extension to antrum/mastoid  5=combined both pars flaccid and pars tensa  stage I: one site  stage II: more than two sites  stage III: a with extracranial complications and/or intratemporal pathologic conditions  stage IV: with intracranial complications |
| Site_ | attic or mesotympanum;  Origin of cholesteatoma | 1= attic origin  2= mesotympanum origin+ combined type |
| Stage_ | site involvement  Extension of cholesteatoma | 1= one site  2= more than one sites/with complication |
| Type_T | type of tympanoplasty | 1=type I  2=type III-m (minor)  3=type III-M (major)  4=type IV(only footplate remained) |
| Type III | Classification of type III | 0= not type III  1= stapes columella  2= minor columella  3= major columella |
| Graft_III | graft using in type III tympanoplasty | 0=not type III  1=incus  2=malleus  3=cortical bone  4=prosthesis |
|  |  |  |
| stapes | stapes condition | 0=present  1=eroded  2=absence of suprastructure |
| stapes_ | stapes condition | 0=present (including eroded)  1=absence of suprastructure |
| incus | Incus condition | 0=present  1=eroded  2=absence |
| malleus | malleus condition | 0=present  1=eroded  2=absence |
| O score | ossicle destruction | 0=normal or destruction of 1 ossicle  1= destruction of 2 ossicles  2= destruction of 3 ossicles |
| tensor | resection of tensor tympani tendon | 0=preserved  1=resected  2=not identified |
| chorda | preservation of chorda tympani, | 0=preserved  1=sacraficed  2=not identified |
| mucosa | Middle ear cavity mucosa status | 0=normal  1=edema  2=granulation  3=adhesive/atalectasis |
| antrum | Antrum patency | 0=patent  1=occluded |
| ET | E tube orifice | 0=patent  1=occluded |
| Recons | Single stage or 2^nd^ stage reconstruction | 1=single stage  2=2^nd^ stage |
| FN | dehiscence of facial n. | 0=no  1=yes |
| LSSC | LSSC fistula | 0=no  1=yes |
| Tegmen | Tegmen tympani/mastoidium dehiscence | 0=no  1=yes |
| Sig | Sigmoid sinus dehisence | 0=no  1=yes |
| OPT | OP time | N (mins) |
|  |  |  |
| FU_3m | Follow up PE finding at 3 months | 0=healed and dry  1=healed but with wax  2=granulation/discharge |
| FU_6m | Follow up PE finding at 6 months | 0=healed and dry  1=healed but with wax  2=granulation/discharge |
| FU_12m | Follow up PE finding at 12 months | 0=healed and dry  1=healed but with wax  2=granulation/discharge |
| ReOP | Receiving revision surgery | 0=no, 1=yes |
| Com | Complication during F/U | 0=no  1= infection with granulation/pus  2= Recidivism with recurrent or residual cholesteatoma  3= Meatal stenosis,  4= CSF leak  5= Facial n. injury,  6= total deafness/SNHL  7= OME  8= drum perforation |
| FUL | Follow up length | N (months) |
| ReSite | Subsite of Recidivism |  |
| Pre_inf | Otorrhea at time of surgery | 0=no otorrhea  1=otorrhea |
| Perforation | Size of perforation | 0=retraction pocket/ atelectasis  1=<50% perforation  2=>50% perforation |
| Perforation_ | Size of perforation | 1=<50% perforation or retraction pocket/ atelectasis  2=>50% perforation |
| Contra | state of contralateral ear | 0=normal  1=OME/atelectasis/perforation |
